# Mental health acts and out-of-hospital care: Legislative boundaries and the paramedic role in Australian mental health legislation

**DOI:** 10.1371/journal.pone.0341138

**Published:** 2026-03-06

**Authors:** Louise Roberts, Stacey Masters, Julie Henderson

**Affiliations:** College of Medicine and Public Health, Flinders University, Adelaide, Australia; All India Institute of Medical Sciences - Raipur, INDIA

## Abstract

**Background:**

Changes to Australian mental health legislation have expanded the role of paramedics in managing mental illness. Paramedics in most Australian states and territories can now temporarily detain and transport people suspected of being mentally ill for further assessment if these people are viewed as being at risk or as posing a risk to others and as lacking capacity for decision making.

**Aim:**

This paper examines current mental health legislation from the eight Australian states and territories. We explore the conditions for transport to involuntary care and additional powers granted to paramedics across jurisdictions to identify the differences and implications of legislation in this space.

**Methods:**

Current Australian mental health legislation was examined for: how mental illness is defined; how capacity is defined; how the Acts refer to paramedics; and the powers granted to paramedics under each Act.

**Results:**

All jurisdictions but Western Australia and Victoria have made legislative changes to authorise paramedics to initiate transport for assessment. Although Victoria has made legislative changes to authorise paramedics to initiate transport for assessment, a subsequent Amendment has paused the introduction of this change. The power to authorise paramedics lies with the head of ambulance services in some states and territories and with senior health officials, e.g., the Chief Psychiatrist in others. Paramedics are required to judge patient capacity and level of risk prior to transport.

**Conclusion:**

Legislation has expanded the role of paramedics working with people with mental illness and guides how paramedics conceptualise, interpret and enact their powers in responding to patients with mental illness.

## Introduction

Higher demand for community and acute mental health services, the associated financial costs, and current workforce shortages have far-reaching consequences for individuals living in the community with mental illness and for society in general [1]. These social and structural changes present the paramedic workforce with changing demands and expectations in service delivery, and greater responsibility for clinical decision-making regarding provision of care and the need for further assessment of persons presenting with mental illness. In Australia there were approximately 310,200 emergency department (ED) occasions of service with a mental health-related principal diagnosis during 2023–24 which equates to approximately 3.4% of all ED occasions of service [2]. These increases in acute mental health presentations have been attributed to factors such as the shift in health care provision from institutional settings (hospital and inpatient units) to emergency departments, an aging population, workforce shortages and the prevalence of chronic conditions [3]. Paramedics play a larger role in filling the gap between immediate care and other health and social services [4]. Paramedics now attend an increasing number of lower acuity and urgent primary care presentations as well as those with complex needs all of which dictate assessment, management and referral decisions [4–6]. The increase in demand and higher needs of those in mental health crisis has seen the development of co-response teams (mental health, police and paramedics) internationally and nationally to provide broader assessment and referral options [7–9]. These changes promoted organisational and structural change including bringing paramedics under the national regulatory banner and recognition as a profession, university-based training, electronic documentation, further training both at graduate and post-graduate levels, and a greater emphasis on evidence-based practice [10].

A growth in use of emergency services and the increasing role for paramedics in responding to mental illness is also reflected in changes to mental health legislation, most notably through the granting of power to involuntarily transport a person suspected of being mentally ill to a designated mental health facility (e.g., Emergency Departments and specific mental health services) for assessment. The objective of this paper is to investigate how paramedics and their role are shaped and defined within current Australian state and territories mental health acts. We explore how paramedics are positioned within mental health legislation. The positioning of paramedics is framed within the definition of mental illness within the acts, the labelling of paramedics, and their role and powers as outlined in the legislation. All will be explored in this paper.

### Legislative context

The states and territories have constitutional responsibility for mental health legislation and providing mental health services in Australia while the Commonwealth government provides funding for the universal health care system (Medicare), aged care and social support and more recently for the National Disability Insurance Scheme [11–12]. The Commonwealth government can indirectly impact mental health legislation through ratification of international treaties or United Nations (UN) covenants.. The Commonwealth has used this power on two occasions through ratification of the United Nations General Assembly’s *Principles for the Protection of People with Mental Illness and for the Improvement of Mental Health Care* [13] released in 1991 and more recently the 2006 *Convention on the Rights of Persons with Disabilities* (CRPD) [14].

Australia ratified the UN CRPD in July 2008. The CRPD differs from previous rights instruments as it addresses civil and political as well as social and economic rights [15]. It adopts a social rather than a medical model of disability shifting the focus from the individual deficits of the person experiencing the disability to systemic deficits and the adaptations required to enable full participation in society [14–17]. Four articles from the CRPD have direct application for mental health. Article 17 protects the integrity of the person on an equal basis with others, Article 14 relates to liberty and security of persons and Article 12 provides for equal recognition before the law [14,17]. A fourth article, Article 25 of the CRPD requires that:

State Parties recognise that persons with disabilities have the right to the enjoyment of the highest attainable standard of health without discrimination on the basis of disability [14,18].

Ratification of the CRPD has required a revision of Australian mental health legislation to bring it in line with the Convention. Mental health legislation in turn, impacts clinical practice. Article 17 of the CRPD states that: “[e]very person with disabilities has a right to respect for his or her physical and mental integrity on an equal basis with others.” [14] McSherry notes that earlier iterations sought to protect people with disabilities including the mentally ill from forced institutionalisation [15]. Article 14 is more explicit. It states in part, that “the existence of a disability shall in no case justify a deprivation of liberty.” [14] This can be interpreted as prohibiting involuntary detention of people with mental illness [19,20]. This interpretation has been widely debated. The European Court for Human Rights (ECHR) for example, has rejected a prohibition on involuntary detention [19]. Minkowitz notes that mental health legislation generally allows involuntary detention adopting a medical rather than a social model of disability [20].

Australian mental health legislation, in line with other countries, enables involuntary detention [17]. As part of their role paramedics can restrain people with presumed mental illness for a limited time for the purpose of medical/psychiatric assessment (involuntary care). A central determinant of detention in Australian mental health legislation is capacity for harm to self or others [21]. A focus upon harm places risk assessment and management at the centre of treatment requiring practitioners to balance patient freedom with clinician and public safety [22–23]. Further, it requires the practitioner judge the potential for harm. Callaghan and Ryan argue that methods for determining future risk are unreliable and may result in denial of treatment to those requiring it and detention of people who may not require it [24].

A second criterion for detention in Australia is that the person has a mental illness or disorder as defined in legislation [25]. Legislation generally reflects current medical understandings of mental illness [11]. Biomedical definitions of mental illness shift focus from the social context of care to diagnosis and impairment, evident in legislation which defines mental illness in terms of behavioural deficits [17], placing greater responsibility on frontline providers of health care to ensure assessment and care of those with mental health concerns.

While Australian mental health legislation enables involuntary detention, the tenet of least restrictive practice is critical to the interpretation of current mental health legislation. The principles that underlie mental health legislation are often fore fronted in the legislation or stated within the legislation as foundational concepts. Least restrictive principles are clearly stated in all state and territory mental health legislation. Paramedics are integral in the first line response and service provision of acute mental health care and are therefore required to consider least restrictive principles in the way they implement restraint and care.

A final criterion addressed by Gray et al. is capacity [25]. Capacity is covered by Article 12 of the CRPD which requires signatories to ensure equal recognition before the law. The second clause states that:

States Parties shall recognize that persons with disabilities enjoy legal capacity on an equal basis with others in all aspects of life [14].

Article 12 recognises legal capacity as both the legal standing of the individual in requiring equal treatment before the law for people with disabilities but also the legal agency of that person [26]. Legal agency requires that states “respect the rights, will and preferences of the person” and ensure that support is available to enable the disabled person to exercise their legal capacity.[14 (p.763), 26] Series notes that a medico-legal definition of mental capacity has been utilised within mental health legislation which associates capacity with a person’s ability to give informed consent to a particular treatment decision [27]. Capacity is usually understood as existing if the person can understand the choices available; weigh up the consequences of their choices and the impact of those consequences; and communicate their decision [28]. Series argues that Article 12 of the CRPD separates legal capacity from mental capacity through recognising the role of environment in enabling or hindering the agency of a person with disabilities, removing the association of capacity with individual deficit [27].

In practice, most mental health legislation utilises a medio-legal definition of capacity focusing upon the functional capacity of the individual [27,29]. This approach is reflected in paramedic practice. Evans et al. for example, associate capacity with taking in and retaining information, believing it and weighing the information to balance risk and needs [30]. In addition to mental health legislation, paramedics are required to have some knowledge of other relevant legislation that deals with consent and refusal of treatment (e.g., *The Consent to Medical Treatment and Palliative Care Act (SA)*) that promote a medico-legal definition of capacity and its relevance to mental health assessment and legislation.

This paper is part of a larger study analysing how policy and practice guidelines for mentally ill patients have been developed and implemented by ambulance services in Australia [31]. Legislation and how it defines mental illness and the paramedic role, links to how policy and care directives are developed, translated and interpreted to frontline staff (paramedics) and enacted in clinical practice. These legislative documents influence how someone in mental distress is approached, assessed and treated, and define how paramedics relate to the broader health care team. Paramedics in most Australian jurisdictions now have the power to take a person to a designated mental health facility involuntarily for assessment for mental illness. This document analysis provides the opportunity to compare the various mental health legislation across Australia as it pertains to paramedic practice, the powers bestowed and how these can affect clinical practice, operational and educational considerations.

## Methods

This paper outlines the results of document analysis of the mental health legislation from the eight Australian state and territory governments. According to Bowen cited in Dalglish “document analysis is a systematic procedure for reviewing or evaluating documents, which can be used to provide context, generate questions, supplement other types of research data, track change over time and corroborate other sources” [32]. There are several ways to approach document analysis and to view documents as social tools that reflect and generate meaning for groups and individuals.

The framework adopted in this paper is the framework proposed by Gorichanaz and Latham [33]. Gorichanaz and Latham’s work frame documents as “experience”. They suggest documents are interactional. They have intent when generated and are given purpose and meaning by how organisations and individuals enact them [33]. In this research, mental health legislation provides the generated intent or “content” and the comparison between mental health acts (the identification of similarities, patterns, or themes) enables discussion on how these similarities and differences define and provide the context for clinical practice for paramedics when they provide care for people presenting with mental health concerns.

For the purposes of this paper the full state and territory name and their acronyms are as follows: Australian Capital Territory (ACT); New South Wales (NSW); Northern Territory (NT); Queensland (QLD); South Australia (SA); Tasmania (Tas); Victoria (Vic) and Western Australia (WA)

The latest version of each Act was accessed online either from the Australasian Legal Information Institute, local State and Territory legislation portals or the health departments of that state or territory. As the *Mental Health Act 2014 (Vi*c) and the *Mental Health Act 2013 (Tas)* were repealed or revised during data collection, the latest Act or version was reviewed to ensure the information was current.

The analysis focused on key areas from the legislation that provide context and influence how paramedics enact clinical care and hence are “experienced” in terms of what paramedics can and cannot do. The decision to transport for assessment for example, depends upon and is influenced by the legal definition of mental illness, perception of risk to self and others and capacity for decision-making of the person transported. All require complex decision-making by the paramedic. Analysis of the legislation for this paper, focuses on four key areas.

1. how mental illness is defined in each Act (including inclusion and exclusion criteria);
2. how capacity is defined in each Act;
3. how the Acts refer to paramedics; and
4. the powers granted to paramedics under each Act.

**Fig 1:**
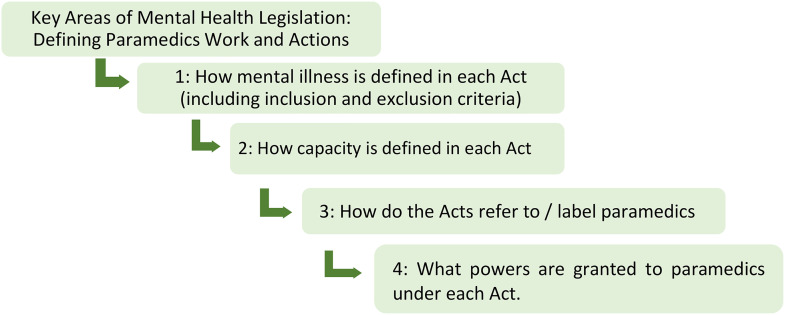
Key questions guiding the analysis of mental health legislation.

These areas were decided upon as a team after discussion and reference to the current literature in this area. The relationship between these key areas from legislation provide context and influence how paramedics enact clinical care is outlined in Fig 1 below.

Templates, comprised of the tables and Figs displayed within this paper, for data extraction and analysis were developed with reference to the areas of focus for this study, current literature and consensus within the research team. State and territory mental health legislation was divided between team members. Each team member completed the relevant sections of the templates from allocated legislation. Regular meetings were conducted to review the data gained from each state and territory mental health legislation as a team. As the data for this paper has been drawn from publicly available legislation ethics approval was not required for this study.

## Results

Analysis centres on the definition of mental illness and exclusionary criteria, the assessment and role of capacity and risk on paramedic practice, how paramedics are labelled and defined within the legislation and the powers they have across jurisdictions.

### How is mental illness defined?

There are subtle differences in how mental illness is conceptualised and framed in legislation across Australian state and territory mental health acts. The following components were common in descriptions of what is deemed as mental illness: impairment, capacity, physical risk, assessment and treatment. Overarching the definitions in the Acts is the concept of impairment, where mental illness is defined as impairment of a person’s mental function (be it temporarily or permanently). Table 1 summarises the definitions adopted in Australian Mental Health Acts.

**Table 1:** Definition of mental illness in Australian Mental Health Acts.

Jurisdiction and legislation	Definition of mental illness	Source, and expanded definition of mental illness
**Australian Capital Territory (ACT)** Mental Health Act 2015 A2015-38	Condition that seriously impairs mental functioning (in one or more areas of thought, mood, volition, perception, orientation or memory).	**Chapter 2.10** **Mental illness** means a condition that seriously impairs (either temporarily or permanently) the mental functioning of a person in 1 or more areas of thought, mood, volition, perception, orientation or memory, and is characterised by— (a) the presence of at least 1 of the following symptoms: (i) delusions; (ii) hallucinations; (iii) serious disorders of streams of thought; (iv) serious disorders of thought form; (v) serious disturbance of mood; or (b) sustained or repeated irrational behaviour that may be taken to indicate the presence of at least 1 of the symptoms mentioned in paragraph (a).
**New South Wales (NSW)**Mental Health Act 2007 No 8.	Temporary or permanent impairment of the mental functioning of a person.	***Chapter 1.4*** ***Mental illness*** *means a condition that seriously impairs, either temporarily or permanently, the mental functioning of a person and is characterised by the presence in the person of any one or more of the following symptoms—* *(a) delusions,* *(b) hallucinations,* *(c) serious disorder of thought form,* *(d) a severe disturbance of mood,* *(e) sustained or repeated irrational behaviour indicating the presence of any one or more of the symptoms referred to in paragraphs (a)–(d).*
**Northern Territory (NT)** Mental Health and Related Service Act 1998 REPM023	Condition which seriously impairs mental functioning (in one or more areas of thought, mood, volition, perception, orientation or memory). Determination made in accordance with international standards	***Part 1.6 – Mental illness*** *(1) A* ***mental illness*** *is a condition that seriously impairs, either temporarily or permanently, the mental functioning of a person in one or more of the areas of thought, mood, volition, perception, orientation or memory and is characterised:* *(a) by the presence of at least one of the following symptoms:* *(i) delusions;* *(ii) hallucinations;* *(iii) serious disorders of the stream of thought;* *(iv) serious disorders of thought form;* *(v) serious disturbances of mood; or* *(b) by sustained or repeated irrational behaviour that may be taken to indicate the presence of at least one of the symptoms referred to in paragraph (a).* *(2) A determination that a person has a mental illness is only to be made in accordance with internationally accepted clinical standards.*
**Queensland (Qld)** Mental Health Act 2016 2016–5	Clinically significant disturbance of thought, mood, perception or memory.	***Part 3.10*** *(1)* ***Mental illness*** *is a condition characterised by a clinically significant disturbance of thought, mood, perception or memory.*
**South Australia (SA)** Mental Health Act 2009 Version: 22.6.2023	Illness or disorder of the mind	***Part 1.3*** ***Mental illness*** *means any illness or disorder of the mind; see also Schedule 1 (Certain conduct may not indicate mental illness);*
**Tasmania (Tas)** Mental Health Act 2013 2013 − 2	Experiences temporarily, repeatedly or continually – a serious impairment of thought, mood, volition, perception and/or cognition.	***Part 2.4*** *(1) For the purposes of this Act –* *(a)* ***a person is taken to have a mental illness if*** *he or she experiences, temporarily, repeatedly or continually –* *(i) a serious impairment of thought (which may include delusions); or* *(ii) a serious impairment of mood, volition, perception or cognition; and* *(b) nothing prevents the serious or permanent physiological, biochemical or psychological effects of alcohol use or drug-taking from being regarded as an indication that a person has a mental illness.*
**Victoria (VIC)** Mental Health and Wellbeing Act 2022 39/2022	Medical condition characterised by a significant disturbance of thought, mood, perception or memory.	***Part 1.2*** ***Mental illness*** *is a medical condition that is characterised by a significant disturbance of thought, mood, perception or memory.*
**Western Australia (WA)** Mental Health Act 2014 024 of 2014	A disturbance of thought, mood, volition, perception, orientation or memory which significantly impairs the person’s judgement or behaviour.	***Division 2.6*** *(1) A person has a mental illness if the person has a condition* *that —* *(a) is characterised by a disturbance of thought, mood, volition, perception, orientation or memory; and* *(b) significantly impairs (temporarily or permanently) the person’s judgment or behaviour.*

Mental Health Act 2015 (ACT); Mental Health Act 2007 (NSW); Mental Health and Related Service Act 1998 (NT); Mental Health Act 2016 (Qld); Mental Health Act 2009 (SA); Mental Health Act 2013 (Tas); Mental Health and Wellbeing Act 2022 (Vic); Mental Health Act 2014 (WA)

Underpinning these Acts are medical and clinical definitions of mental illness that are most commonly expressed in terms of the presence of a range of symptoms that are clinically significant or have an effect on the person’s behaviour or ability to function. These symptoms include sustained or repeated irrational behaviour, and disturbance of thought, mood, perception or memory. Clinical standards are also referenced in several of the Acts (notably QLD & NT). The reference to clinical standards and clinical significance are relevant in that they assume a level of knowledge of current mental health clinical standards and clinically significant findings from the mental state assessment and examination Table 2.

**Table 2:** Exclusionary Criteria.

Jurisdiction	Exclusionary Criteria
**Australian Capital Territory (ACT)** Mental Health Act 2015 A2015-38	**ACT Mental Health Act 2015, Part 11** 11) People not to be regarded as having mental disorder or mental illness For this Act, a person is not to be regarded as having a mental disorder or mental illness only because of any of the following: (a) **the person expresses or refuses or fails to express, or has expressed or has refused or failed to express**, a particular political opinion or belief; (b) religious opinion or belief; (c) a particular philosophy; (d) a particular sexual preference or sexual orientation; (e) **the person engages in or refuses or fails to engage in, or has engaged in or has refused or failed to engage in,** a particular political activity; (f) a particular religious activity; (g) **the person engages in or has engaged in** sexual promiscuity; (h) immoral conduct; (i) illegal conduct; (j) the person takes or has taken alcohol or any other drug; (k) antisocial behaviour.
**New South Wales (NSW)** Mental Health Act 2007 No 8.	**Mental Health Act 2007 (NSW), s 16, Chapter 3, Part 1.** **16) Certain words or conduct may not indicate mental illness or disorder** (cf 1990 Act, s 11) (1) A person is not a mentally ill person or a mentally disordered person merely because of any one or more of the following— (a) **the person expresses or refuses or fails to express or has expressed or refused or** **failed to express a** particular political opinion or belief, (b) particular religious opinion or belief, (c) particular philosophy, (d) particular sexual preference or sexual orientation, (e) particular political activity, (f) particular religious activity, (g) **the person engages in or has engaged in** a particular sexual activity or sexual promiscuity, (h) immoral conduct, (i) illegal conduct, (l) anti-social behaviour, (j) the person has an intellectual disability or developmental disability, (k) the person takes or has taken alcohol or any other drug, (m) the person has a particular economic or social status or is a member of a particular cultural or racial group. (2) Nothing in this Part prevents, in relation to a person who takes or has taken alcohol or any other drug, the serious or permanent physiological, biochemical or psychological effects of drug taking from being regarded as an indication that a person is suffering from mental illness or other condition of disability of mind.
**Northern Territory (NT)** Mental Health and Related Service Act 1998 REPM023	**Mental Health and Related Services Act 1998 (NT), s 6(3)** (3) A person is not to be considered to have a mental illness merely because he or she: (a**) expresses or refuses or fails to express a** particular political or religious opinion or belief, a particular philosophy or a particular sexual preference or sexual orientation; or (b) **engages, or refuses or fails to engage, in** a particular political, religious or cultural activity; or (c) engages, or has engaged, in sexual promiscuity, immoral or illegal conduct or anti-social behaviour; or (d) has a sexual disorder; or (e) is intellectually disabled; or (f) uses alcohol or other drugs; or (g) has a personality disorder or a habit or impulse disorder; or (h) has, or has not, a particular political, economic or social status; or (j) communicates, or refuses or fails to communicate, or behaves or refuses or fails to behave, in a manner consistent with his or her cultural beliefs, practices or mores; or (k) is, or is not, a member of a particular cultural, racial or religious group; or (m) is involved, or has been involved, in family or professional conflict; or (n) has been treated for mental illness or has been detained in a hospital that provides treatment of mental illness; or (p) has been admitted as an involuntary patient on the grounds of mental disturbance or complex cognitive impairment; or (q) has acquired brain damage.
**Queensland (Qld)** Mental Health Act 2016 2016–5	**Queensland Mental Health Act 2016, s 10(2)(3)(4)** (2) However, a person must not be considered to have a mental illness merely because— (a) **the person holds or refuses to hold a particular** religious, cultural, philosophical or political belief or opinion; or (b) the person is a member of a particular racial group; or (c) the person has a particular economic or social status; or (d) the person has a particular sexual preference or sexual orientation; or (e) the person engages in sexual promiscuity; or (f) the person engages in immoral or indecent conduct; or (g) the person takes drugs or alcohol; or (h) the person has an intellectual disability; or (i) the person engages in antisocial behaviour or illegal behaviour; or (j) the person is or has been involved in family conflict; or (k) the person has previously been treated for a mental illness or been subject to involuntary assessment or treatment. (3) Subsection (2) does not prevent a person mentioned in the subsection having a mental illness. *Examples for subsection (3)—* 1 A person may have a mental illness caused by taking drugs or alcohol. 2 A person may have a mental illness as well as an intellectual disability. (4) A decision that a person has a mental illness must be made in accordance with internationally accepted medical standards.
**South Australia (SA)** Mental Health Act 2009 Version: 22.6.2023	**Mental Health Act 2009 (SA), Schedule 1** **Schedule 1—Certain conduct may not indicate mental illness** A person does not have a mental illness merely because of any 1 or more of the following: (a) **the person expresses or refuses or fails to express, or has expressed or refused or failed to express, a** particular political opinion or belief; (b) particular religious opinion or belief; (c) particular philosophy; (d) particular sexual preference or sexual orientation; (e) **the person engages in or refuses or fails to engage in, or has engaged in or refused or failed to engage in,** a particular political activity;(f) a particular religious activity; (g) particular sexual activity or sexual promiscuity; (h) **the person engages in or has engaged in** immoral conduct; (i) illegal conduct; (l) anti-social behaviour; (j) the person has developmental disability of mind; (k) the person takes or has taken alcohol or any other drug; (m) the person has a particular economic or social status or is a member of a particular cultural or racial group. However, nothing prevents, in relation to a person who takes or has taken alcohol or any other drug, the serious or permanent physiological, biochemical or psychological effects of drug taking from being regarded as an indication that a person is suffering from mental illness.
**Tasmania (Tas)** Mental Health Act 2013 2013 − 2	**Mental Health Act 2013, s 4(2) (Part 2)** (2) However, under this Act, a person is not to be taken to have a mental illness by reason only of the person’s – (a) **current or past expression of, or failure or refusal to express, a** particular political opinion or belief; or (b) particular religious opinion or belief; or (c) particular philosophy; or (d) particular – (i) sexual preference or orientation; or (ii) gender identity or expression; or (e) particular political or religious activity; or (f) **current or past engagement in** a particular sexual activity or sexual promiscuity; or (g) illegal conduct; or (h) an antisocial activity; or (i) particular economic or social status; or (j) membership of a particular cultural or racial group; or (k) intoxication (however induced); or (l) intellectual or physical disability; or (m) acquired brain injury; or (n) dementia; or (o) temporary unconsciousness
**Victoria (VIC)** Mental Health and Wellbeing Act 2022 39/2022	**Mental Health and Wellbeing Act 2022, s 4 (2) (3) (Part 1.2)** **4) Meaning of mental illness in this Act** (2) A person is not to be considered to have mental illness by reason only of any one or more of the following— (a) **that the person expresses or refuses or fails to express a** particular political opinion or belief; (b) particular religious opinion or belief; (c) particular philosophy; (d) that the person expresses or refuses or fails to express a particular sexual preference, gender identity or sexual orientation; (e) **that the person engages in or refuses or fails to engage in** a particular political activity; (f) a particular religious activity; (g) that the person has engaged in a certain pattern of sexual behaviour; (h) that the person engages in conduct that is contrary to community standards of acceptable conduct; (i) that the person engages in illegal conduct; (j) that the person engages in antisocial behaviour; (k) that the person is intellectually disabled; (l) that the person uses drugs or alcohol; (m) that the person has a particular economic or social status or is a member of a particular cultural or racial group; (n) that the person is or has previously been involved in family conflict; (o) that the person is experiencing or has experienced psychological distress; (p) that the person has previously been diagnosed with, or treated for, mental illness. (3) Subsection (2)(l) does not prevent the serious temporary or permanent physiological, biochemical or psychological effects of using drugs or alcohol from being regarded as an indication that a person has mental illness.
**Western Australia (WA)** Mental Health Act (2014)2014 024 of 2014	***Mental Health Act 2014, s 6 (2) (3) (Part 2 Division 2)*** (2) A person does not have a mental illness merely because one or more of these things apply — (a) **the person holds, or refuses or fails to hold, a** particular religious, cultural, political or philosophical belief or opinion; (b) the person engages in, or refuses or fails to engage in, a particular religious, cultural or political activity; (c) the person is, or is not, a member of a particular religious, cultural or racial group; (d) the person has, or does not have, a particular political, economic or social status; (e) the person has a particular sexual preference or orientation; (f) the person is sexually promiscuous; (g) the person engages in indecent, immoral or illegal conduct; (h) the person has an intellectual disability; (i) the person uses alcohol or other drugs; (j) the person is involved in, or has been involved in, personal or professional conflict; (k) the person engages in anti-social behaviour; (l) the person has at any time been — (i) provided with treatment; or (ii) admitted by or detained at a hospital for the purpose of providing the person with treatment. (3) Subsection (2)(i) does not prevent the serious or permanent physiological, biochemical or psychological effects of the use of alcohol or other drugs from being regarded as an indication that a person has a mental illness.

Mental Health Act 2015 (ACT); Mental Health Act 2007 (NSW); Mental Health and Related Service Act 1998 (NT); Mental Health Act 2016 (Qld); Mental Health Act 2009 (SA); Mental Health Act 2013 (Tas); Mental Health and Wellbeing Act 2022 (Vic); Mental Health Act 2014 (WA)

All Acts also include a range of exclusionary criteria which provide legal protection against coercive treatment. The exclusionary criteria reflect the civil rights protections outlined in international human rights instruments [11].

The exclusionary criteria are common across all jurisdictions and address the following key human rights. A person cannot be judged as being mentally ill on the basis of: expression of a particular political opinion or belief or activity; religious opinion or belief or activity; particular philosophy; sexual preference or orientation or activity; engagement in illegal conduct or antisocial activity; particular economic or social status; membership of a particular cultural or racial group; intoxication [drug and alcohol] (however induced); intellectual or physical disability; acquired brain injury or dementia. The definitions of mental illness and exclusionary criteria impact paramedic practice. The definitions are often broad and provide scope for interpretation of when behaviour allows paramedics to enact their powers under the Act to transport for further medical assessment and treatment. This opens paramedic clinical practice to wide interpretation and implementation and arguably relies on appropriate training and knowledge base in this area.

Risk is a prerequisite for involuntary care and transport for further assessment in all mental health Acts. The use of involuntary care may be made for the purpose of protection from harm (including self-harm and the risk of mental or physical deterioration) and protection of others from harm. Risk is also referred to more broadly within the Acts. This broader focus refers to the benefit or risks of treatment and management, the need for monitoring and continual review and to ensure that any intervention is not detrimental but therapeutic in nature Table 3. These broader references to risk pertain to mental health facilities, psychiatric assessment and management and not to the pre-hospital environment and specifically paramedic, scope and clinical practice. The following table outlines where in the legislation risk is referred to in relation to paramedic role and powers.

**Table 3:** Risk: Harm to Self, Others and/ or the need for Medical Review.

Jurisdiction	Risk: Harm to Self, Others and/ or the need for Medical Review
**Australian Capital Territory (ACT)** Mental Health Act 2015 A2015-38	**ACT Mental Health Act 2015, s 80** (1) A police officer or authorised ambulance paramedic may apprehend a person if the police officer or paramedic believes on reasonable grounds that— (a) the person has a mental disorder or mental illness; and *(b) the person has attempted or is likely to attempt—* *(i) suicide; or* *(ii) to inflict serious harm on the person or another person; and* *(c) the person—* *(i) requires an immediate examination by a doctor; and* *(ii) does not agree to be examined immediately.* *Note* See s 263 (Powers of entry and apprehension) and s 264 (Powers of search and seizure). (2) In forming a belief about a person for subsection (1), a police officer or authorised ambulance paramedic is not required to make a medical assessment or clinical judgement about the person.
**New South Wales (NSW)** Mental Health Act 2007 No 8.	**Mental Health Act 2007 (NSW), s 13, 14, 15, Chapter 3, Part 1.** **13 Criteria for involuntary admission etc as mentally ill person or mentally disordered person** A person is a mentally ill person or a mentally disordered person for the purpose of— (a) the involuntary admission of the person to a mental health facility or the detention of the person in a facility under this Act, or (b) determining whether the person should be subject to a community treatment order or be detained or continue to be detained involuntarily in a mental health facility, if, and only if, the person satisfies the relevant criteria set out in this Part. **14 Mentally ill persons** (1) A person is a mentally ill person if the person is suffering from mental illness and, owing to that illness, there are reasonable grounds for believing that care, treatment or control of the person is necessary— *(a) for the person’s own protection from serious harm, or* *(b) for the protection of others from serious harm.* *(2) In considering whether a person is a mentally ill person, the continuing condition of the person, including any likely deterioration in the person’s condition and the likely effects of any such deterioration, are to be taken into account.* **15 Mentally disordered persons** A person (whether or not the person is suffering from mental illness) is a mentally disordered person if the person’s behaviour for the time being is so irrational as to justify a conclusion on reasonable grounds that temporary care, treatment or control of the person is necessary— *(a) for the person’s own protection from serious physical harm, or* *(b) for the protection of others from serious physical harm.*
**Northern Territory (NT)** Mental Health and Related Service Act 1998 REPM023	**Mental Health and Related Services Act 1998 (NT) s 31** **31 Detention by paramedic** (1) A paramedic may detain a person being conveyed in an ambulance for up to 6 hours where the paramedic believes, on reasonable grounds, that the person may fulfil the criteria for involuntary admission on the grounds of mental illness or mental disturbance. (2) When detaining a person under subsection (1), a paramedic may use reasonable measures, including the use of restraints, on the person: (a) to prevent the person *causing serious harm to the person or to someone else; or* *(b) to prevent behaviour of the person likely to cause serious harm to the person or to someone else; or* *(c) to prevent further physical or mental deterioration of the person; or* *(d) to relieve acute symptomatology.*
**Queensland (Qld)** Mental Health Act 2016 2016–5	**Queensland Mental Health Act 2016, Chapter 1, Section 12** **12 Meaning of *treatment criteria*** (1) The ***treatment criteria*** for a person are all of the following— (a) the person has a mental illness; (b) the person does not have capacity to consent to be treated for the illness; (c) because of the person’s illness, the absence of involuntary treatment, or the absence of continued involuntary treatment, is likely to result in— (i) imminent serious harm to the person or others; or (ii) the person suffering serious mental or physical deterioration. (2) For subsection (1)(b), the person’s own consent only is relevant. (3) Subsection (2) applies despite the *Guardianship and* *Administration Act 2000*, the *Powers of Attorney Act 1998* or any other law.
**South Australia (SA)** Mental Health Act 2009 Version: 22.6.2023	**Mental Health Act 2009 (SA) s 56** **56—Powers of authorised officers relating to persons who have or appear to have mental illness** (1) This section applies to a person if— (a) an authorised officer believes on reasonable grounds that the person is a patient in respect of whom— (i) a patient assistance request has been issued under section 54A(1); or (ii) a patient transport request has been issued under section 55(1); or (b) an authorised officer believes on reasonable grounds that the person is a patient who is absent without leave; or (c) it appears to an authorised officer that— (i) the person has a mental illness; and (ii) the person has caused, or there is a *significant risk of the person causing, harm to himself or herself or others or property or the person otherwise requires medical examination.* (2) An authorised officer may form an opinion about a person under subsection (1)(c) based on the officer’s observations of the person’s behaviour or appearance or reports about the person’s behaviour, appearance or history (which may include reports about matters occurring outside the State).
**Tasmania (Tas)** Mental Health Act 2013 2013 − 2	**Mental Health Act 2013, s 25 (Part 3)** 25. Assessment criteria The assessment criteria are – (a) the person has, or appears to have, *a mental illness that requires or is likely to require treatment for –* (*i) the person’s health or safety; or* *(ii) the safety of other persons; and* (b) the person *cannot be properly assessed with regard to the mental illness* or the making of a treatment order except under the authority of the assessment order; and (c) the *person does not have decision-making capacity*.
**Victoria (VIC)** Mental Health and Wellbeing Act 2022 39/2022	**Mental Health and Wellbeing Act 2022, s 232 (1a, 1b) (Part 5.2)** **Part 5.2—Power to take a person into care and control in a mental health crisis** **232 Taking a person into care and control in a mental health crisis** (1) An authorised person who is a police officer, a protective services officer or a member of a prescribed class of persons may take a person into care and control under this section if the authorised person is satisfied that— (a) the person appears to have mental illness; and (b) because of the person’s apparent mental illness, it is necessary to take the person into *care and control to prevent imminent and serious harm to the person or to another person.* (2) A person remains in an authorised person’s care and control under this section until the person’s care and control ends in accordance with section 239.
**Western Australia (WA)** Mental Health Act 2014 024 of 2014	* Paramedics are not authorised to instigate involuntary care but can transport a person if a transport order has been provided by a medial practitioner. It is the police’s role to apprehend and assist staff to transport. Risk is outlined for police as risk to self, others and property. **Mental Health Act 2014 (WA), Part 10, s 149** **149. Operation of transport order** (1) A transport order made in respect of a person authorises a transport officer or, if subsection (2) applies, a police officer to do these things — (a) apprehend the person and, for that purpose, exercise the powers under sections 159(2) and 172; (b) if the person is apprehended — transport the person to the hospital or other place specified in the order as soon as practicable and, in any event, before the transport order expires; (c) for the purpose of transporting the person, detain the person until the first of these things occurs — (i) the person is received into the hospital or other place; (ii) the transport order expires. **Mental Health Act 2014 (WA), Part 11, Division 1, s 156** **Part 11 — Apprehension, search and seizure powers** **Division 1 — Apprehension powers** **156. Apprehension by police officer of person suspected of** **having mental illness** (1) A police officer may apprehend a person if the officer reasonably suspects that the person — (a) *has a mental illness*; and (b) because of the mental illness, needs to be apprehended to — (i) *protect the health or safety of the person or the* *safety of another person; or* (ii) prevent the person *causing, or continuing to* *cause, serious damage to property.*

Mental Health Act 2015 (ACT); Mental Health Act 2007 (NSW); Mental Health and Related Service Act 1998 (NT); Mental Health Act 2016 (Qld); Mental Health Act 2009 (SA); Mental Health Act 2013 (Tas); Mental Health and Wellbeing Act 2022 (Vic); Mental Health Act 2014 (WA)

Capacity is also referenced in all Australian mental health Acts. Impairment of a person’s mental function may affect their capacity for decision making and consent and is a factor in whether a person refusing treatment can be transported by paramedics for assessment. Capacity is defined in most mental health Acts in relation to understanding, retaining, and weighing information and communicating decisions. If the person is viewed as having capacity, they cannot be transported if they refuse treatment.

Table 4 outlines the definition of capacity in the mental health Acts. All Acts require assessment of capacity for decision making however, the older Acts (NT and NSW) do not provide a specific or operational definition of capacity. While the NT legislation identifies broad areas in which cognitive impairment may be evidenced (including decision making, problem solving and communication) the Act does not specify criteria for determination of capacity.

**Table 4:** Criteria for determining capacity in current Australian mental health legislation.

	ACT	NSW	NT	Qld	SA	Tas	Vic	WA
Presumption of capacity	√	√	√	√	√	√	√	√
**Criteria for lack of capacity**								
Understanding information	√			√	√	√	√	√
Retaining information					√	√	√	√
Using and weighing information	√			√	√	√	√	√
Communicating decisions	√			√	√	√	√	√

Shaded cells denote the absence of a criterion for determining capacity.

Mental Health Act 2015 (ACT); Mental Health Act 2007 (NSW); Mental Health and Related Service Act 1998 (NT); Mental Health Act 2016 (Qld); Mental Health Act 2009 (SA); Mental Health Act 2013 (Tas); Mental Health and Wellbeing Act 2022 (Vic); Mental Health Act 2014 (WA)

### How are paramedics defined?

Paramedics are broadly described using the term Ambulance Officer and are generally designated under the various state and territory Acts as an Authorised Officer or Authorised Person. Western Australia appears to be the exception with ambulance personnel identified under the term transport officer. An Ambulance Officer (AO) is generally defined as someone employed as an AO or a volunteer AO in an (approved) organisation that provides ambulance services. Ambulance Officers are authorised by various entities (e.g., the CEO of the SA Ambulance Service, the NSW Director General, and the Chief Health Officer in the Northern Territory) to exercise the powers conferred to them within the Acts. Victoria, Australian Capital Territory and Northern Territory are the only mental health acts that specifically use the term paramedic within the Act. The differing definitions of paramedics in the Mental Health Acts suggest that the recently regulated profession is still being recognised for the changing nature of paramedic work and scope of practice. Paramedics work in a variable and autonomous environment which means that clinical training and decision-making are critical to how they translate and interpret their legislative responsibilities. In providing mental health care paramedics act as first responders meeting the acute need of people in psychological distress and assessing and managing crisis in its initial stages. Although the powers under legislation for paramedics are similar to those of other health professionals such as mental health nurses and social workers the boundaries of clinical practice are different Table 5. These differing roles define boundaries and how paramedics coordinate, refer to and use mental health services or other allied health providers. Paramedics with increasing scope of practice are also contending with the overarching aims of maintaining the person in their home and hospital avoidance which adds to the need for paramedics to assess and make clinical decisions in this space.

**Table 5:** How are paramedics defined in Australian Mental Health Acts.

Jurisdiction	Terminology	Designated as	Authorised by
ACT (2015)	Ambulance paramedic	Authorised officer	Chief Officer, Ambulance Service
NSW (2007)	Ambulance officer	Authorised ambulance officer	Secretary, NSW Health Service
NT (1998)	Paramedic	Designated mental health practitioner*	CEO St John’s Ambulance NT
Qld (2016)	Ambulance officer	Authorised ambulance officer	Appointed under the Ambulance Service Act 1991
SA (2009)	Ambulance officer	Authorised Officer	CEO, SA Ambulance Service
Tas (2013)	Ambulance officer	Mental health officer	Chief Psychiatrist
VIC (2022)	Registered paramedic	Authorised person	Chief Psychiatrist
WA (2014)	–	Transport officers/ but legislation doesn’t include or reference	Chief Psychiatrist Paramedics can apprehend and transport patients if a transport order is completed by a medical practitioner or authorised mental health practitioner

*This appointment requires not less than 2 years approved clinical experience; and successful completion of an approved training and orientation course.

Mental Health Act 2015 (ACT); Mental Health Act 2007 (NSW); Mental Health and Related Service Act 1998 (NT); Mental Health Act 2016 (Qld); Mental Health Act 2009 (SA); Mental Health Act 2013 (Tas); Mental Health and Wellbeing Act 2022 (Vic); Mental Health Act 2014 (WA)

### Paramedics’ role and powers

Paramedics have various roles and powers within the Mental Health Acts. These roles and powers relate to the ways in which mental illness and paramedics themselves are defined in the Acts. The powers held by paramedics are encompassed by what Llewelyn et al.[23(p. 1037)] designate as “involuntary assessment” which they define as “a statutory authority which allows police officers, paramedics and health practitioners to override a person’s autonomy where they form the view that person has a mental illness, and may be at risk of harming themselves, or others”. Assessment and care occur within the context of least restrictive practice, where paramedics are to use these measures only when no other alternative is available. Table 6 outlines where the principle of least restrictive practice is mentioned within each state and territory Act as it pertains to the enacting of powers for paramedics.

**Table 6:** Least Restrictive Principles mentioned in Australian Mental Health Acts.

Jurisdiction: Section/ Part – Least Restrictive Practice	Key principles
**Australian Capital Territory (ACT)** Mental Health Act 2015	**ACT Mental Health Act 2015, s 5(c) (Object 2)** (c) ensure that people with a mental disorder or mental illness receive assessment and treatment, care or support in a way that is least restrictive or intrusive to them;
**New South Wales (NSW)** Mental Health Act 2007 No 8	**New South Wales Mental Health Act 2014, s 68(a)** (a) people with a mental illness or mental disorder should receive the best possible care and treatment in the least restrictive environment enabling the care and treatment to be effectively given
**Northern Territory (NT)** Mental Health and Related Service Act 1998 REPM023	**Mental Health Act 1998 (NT), s. 8(1)(a)** (a) a person who has a mental illness receives the best possible care and treatment in the least restrictive and least intrusive environment enabling the care and treatment to be effectively given;
**Queensland (Qld**) Mental Health Act 2016 2016–5	**Mental Health Act 2016 (Qld), s 3(2) & (3)** (2) The main objects are to be achieved in a way that— (a) safeguards the rights of persons; and (b) is the least restrictive of the rights and liberties of a person who has a mental illness; and (c) promotes the recovery of a person who has a mental illness, and the person’s ability to live in the community, without the need for involuntary treatment and care. (3) For subsection (2)(b), a way is the least restrictive of the rights and liberties of a person who has a mental illness if the way adversely affects the person’s rights and liberties only to the extent required to protect the person’s safety and welfare or the safety of others.
**South Australia (SA)** Mental Health Act 2009 Version: 22.6.2023	**Mental Health Act 2009 (SA), s 7(1)(b)** (b) mental health services should be provided on a voluntary basis as far as possible, and otherwise in the least restrictive way and in the least restrictive environment that is consistent with their efficacy and public safety, and at places as near as practicable to where the patients, or their families or other carers or supporters, reside;
**Tasmania (Tas)** Mental Health Act 2013 2013–2	**Mental Health Act 2013 (Tas), s 12(d)** (d) to provide for such assessment and treatment to be given in the least restrictive setting consistent with clinical need, legal and judicial constraints, public safety and patient health, safety and welfare; **Tasmanian Mental Health Act 2013, Schedule 1, section 15, section 156, section 162, section 228, subsection 1(b)** (b) to interfere with or restrict the rights of persons with mental illness in the least restrictive way and to the least extent consistent with the protection of those persons, the protection of the public and the proper delivery of the relevant service;
**Victoria (VIC)** Mental Health and Wellbeing Act 2022 39/2022	**Mental Health and Wellbeing Act 2022 (Vic), s 12(e)** (e) to protect and promote the human rights and dignity of people living with mental illness by providing them with assessment and treatment in the least restrictive way possible in the circumstances; **Mental Health and Wellbeing Act 2022 (Vic), s 18** Mental health and wellbeing services are to be provided to a person living with mental illness or psychological distress with the least possible restriction of their rights, dignity and autonomy with the aim of promoting their recovery and full participation in community life. The views and preferences of the person should be key determinants of the nature of this recovery and participation. **Mental Health and Wellbeing Act 2022 (Vic), Chapter 5, Part 5.1, s 230** **230 Least restrictive approach principle** So far as is reasonably practicable in the circumstances, the exercise of a power by an authorised person under this Chapter is to be exercised in the least restrictive way possible.
**Western Australia (WA)** Mental Health Act 2014 024 of 2014	**Mental Health Act 2014 (WA), Part 6, Division 2, s 48.** **48. How assessment must be conducted** (1) The assessment must be conducted in the least restrictive way, and the least restrictive environment, practicable. **Western Australia Mental Health Act 2014, Schedule 1, Principle 4, s. 11, 12, 320(2)(f), 333(3)(d), and 351(1)(b)** A mental health service must be easily accessible and safe and provide people experiencing mental illness with timely treatment, care and support of high quality based on contemporary best practice to promote recovery in the least restrictive manner that is consistent with their needs.

Mental Health Act 2015 (ACT); Mental Health Act 2007 (NSW); Mental Health and Related Service Act 1998 (NT); Mental Health Act 2016 (Qld); Mental Health Act 2009 (SA); Mental Health Act 2013 (Tas); Mental Health and Wellbeing Act 2022 (Vic); Mental Health Act 2014 (WA)

Six main powers, all of which focus on measures of involuntary care, are defined in the Acts, including: transport, remain in place, restraint, force, search and use of drugs for chemical restraint. Table 7 shows how these powers are conferred on paramedics within each state. The power to transport a person who has, or is suspected of having, a mental illness is a key power of paramedics in the Acts, with all Acts affording paramedics this power. The person is generally transported to the nearest approved treatment facility or, if that is not possible, to the nearest hospital. In most jurisdictions paramedics can make a clinical judgement that a person needs to be assessed however, in Western Australia the *Mental Health Act 2014* requires that a medical practitioner or authorised mental health practitioner initiate transport for assessment and in Victoria the *Mental Health Act 2022* makes police and Protective Service Officers who police transport hubs, responsible for care and control in a mental health crisis.

**Table 7:** Paramedic powers as outlined in legislation in relation to detention and restraint across Australian jurisdictions.

Jurisdiction	Apprehension for assessment	Search	Enter and remain in place	Use of physical restraint	Use of mechanical restraint	Use of chemical restraint
ACT	Can apprehend and transport for assessment	yes	yes	yes	yes	yes
NSW	Can apprehend and transport for assessment	yes	no (police only)	yes	yes	yes
NT	Paramedics as designated mental health practitioners can detain people for up to 6 hours for assessment	Not specified	yes	yes	yes	yes
Qld	Can apprehend and transport for assessment	Not specified	If an occupier consents; or it is a public place; or a warrant has been issued.	yes	With approval of Chief psychiatrist	Administered by Dr or RN prior to transport
SA	Paramedics as authorised officers can apprehend and transport for further assessment	yes	yes	yes	yes	Yes, if within scope of practice
Tas	Paramedics designated as mental health officers can take people in for assessment	yes	yes	yes	yes	yes
Vic	Registered paramedics as authorised health professionals can transport for further assessment but cannot take a person into care and control in a mental health crisis.	yes	yes	yes	yes	Yes, if directed by a registered medical professional, or within scope of practice.
WA	Can apprehend if a transport order is completed by an authorised mental health practitioner but cannot initiate apprehension	Yes, if a transport order is in effect.	Yes, if a transport order is in effect	yes	Not specified	Yes, if directed by a registered medical professional, or within scope of practice.

Mental Health Act 2015 (ACT); Mental Health Act 2007 (NSW); Mental Health and Related Service Act 1998 (NT); Mental Health Act 2016 (Qld); Mental Health Act 2009 (SA); Mental Health Act 2013 (Tas); Mental Health and Wellbeing Act 2022 (Vic); Mental Health Act 2014 (WA)

The powers of restraint and use of force are the second most common powers conferred on paramedics. The Queensland Ambulance Service identify three forms of restraint [34]. Physical restraint involves the use of part of another’s body, mechanical restraint involves the use of device or equipment and chemical restraint the provision of sedation [34]. The use of force and restraints is supported to prevent harm to the person or others and to prevent further physical and mental deterioration. All states and territories have the power to initiate restraint except WA. Paramedics in WA cannot directly instigate physical and mechanical restraint under the *Mental Health Act 2014 (WA)* unless authorised by a medical practitioner or under the Public Health Act. The power to use drugs for chemical restraint (if it is in the paramedics’ scope of practice) is present in six of the Acts (NSW, SA, Tas, Vic, ACT and NT).

Paramedics in the ACT, NSW, SA, Tas and Vic also are granted the power to search the person and take possession of anything that may cause harm to the person, others or property. In WA, police and transport officers can search the person if safe to do so and a transport order is in effect while in NT and Qld the power to search the person is not specified in the Mental Health Act. Five Acts (ACT, NT, SA, Tas and Vic) also discuss the power to enter into and remain in a place where they suspect the person may be found (e.g., if a transport request has been issued). In NSW and WA, only police can enter and remain in place. In WA a transport officer can enter but only if a transport order is in effect and it is safe to do so. Therefore, in general the responsibility tends to fall to the police to search, enter and apprehend. Paramedics in Qld require consent of an occupier to enter premises, (unless it is a public place and during the hours of operation) or a warrant has been issued.

## Discussion

This paper has described the role of paramedics within mental health legislation and paramedic powers as defined within legislation. The key areas of mental health legislation that define and influence how paramedics are framed and guide care for presentation of mental illness are 1) how paramedics are identified and how their role is defined in legislation; 2) how mental illness is defined; and 3) how legislation frames risk and capacity. These key areas have influence over how paramedics enact their clinical practice (their powers under legislation) and make decisions specifically dealing with presenting cases of suspected mental ill health. The comparisons and key features in mental health legislation, as shown in the results, underpin policy, clinical guidelines, and operating procedures within Ambulance Services nationally.

### How paramedics are identified: Their role and responsibilities

All mental health Acts except the *Mental Health Act 2014* (WA) identify paramedics or ambulance officers within their legislation. This is key as it denotes the significant role paramedics have in providing care for those with mental illness and legislates those responsibilities as a health concern and part of the paramedic role [24]. Safe transport is identified as the primary responsibility of paramedics, but the legislation in several states and territories also confers responsibility for decision-making to enact temporary detention to transport patients for further assessment. This is at odds with other countries such as the United Kingdom, where this responsibility lies with the police [9]. In all states and territories other than Western Australia and Victoria paramedics have been granted this power. As part of being granted these powers paramedics are required to assess and make decisions around key areas impacting the appropriateness of involuntary care and further assessment namely: the definition of mental illness which guides what they recognise and assess as mental illness; assessment of risk; capacity to make informed treatment decisions and the use of restraint. In general, physical, mechanical and chemical restraint can be used in all jurisdictions except Queensland which prevents use of mechanical restraint, but there are restrictions based on the requirement for authorisation and clinical scope of practice.

### Defining mental illness

Australian mental health legislation adopts a view of mental illness based upon deficits of the individual rather than the social model of disability underpinning the CRPD. This approach is in line with legislation in other countries which seeks to protect people with mental illness from unnecessary custodial care through tightening criteria for detention [17,19]. All Mental Health Acts except the *Mental Health Act 2009 (SA)* define mental illness as a disorder of thought, mood and perception (e.g., delusions, hallucinations) with many also including disturbed behaviour. South Australian legislation adopts a broader definition of mental illness ‘as any illness or disorder of the mind’ [25]. Fistein et al. argue that broader definitions of mental illness ensure that people requiring treatment receive it [35]. This may enable access to services meeting Article 25 of the CRPD however, broader definitions require greater discretion on the part of clinicians in relation to what constitutes mental illness and potentially opens the Act to misuse.

Inclusion and exclusion criteria for mental illness within legislation are used as a means of preventing inappropriate involuntary care. The exclusion criteria reflect civil rights protections offered in international treaties and current community values [11]. All Australian mental health Acts include exclusionary criteria relating to political, religious, philosophical and sexual beliefs and practices. People experiencing disorders with identifiable physical causes such as brain injury, dementia and/or developmental or intellectual disability are also excluded. Economic and social status are exclusionary criteria in all states and territories except the Australian Capital Territory; a previous history of treatment for mental illness is an exclusionary criterion in Queensland, Northern Territory, Victoria and Western Australia and family conflict is an exclusionary criterion in Queensland, Northern Territory and Victoria. Tasmanian and Victorian legislation cites gender identity and expression as an exclusionary criterion.

### Capacity and risk

All Mental Health Acts require assessment of capacity and risk be undertaken prior to transportation for further assessment and care. A perception that the person poses a risk to self or others alongside of lack of capacity are required for transport for involuntary assessment. Paramedics utilise a medico-legal definition of capacity. Six of the eight mental health Acts examined provide a definition of capacity based upon assessment of level of mental function. This is at odds with the definition of legal capacity evident in the CRPD but provides some protection against unnecessary detention through provision of guidelines [26]. In two jurisdictions (NSW and NT) operational definitions of capacity are lacking. The absence of criteria for determination of capacity requires paramedics (and other designated mental health professionals) to make a judgement call about a person’s capacity that they may feel ill-equipped for, and which may be difficult to defend in the absence of a standardised assessment. Further, due to the acute or cyclic nature of mental illness, especially when attended to in the community, capacity can be difficult to assess. Key difficulties with assessment of capacity in community or acute settings relate to time constraints and access to past history required to ascertain the capacity or needs of an individual.

Risk is universally associated with risk to self and others and the need for further medical review. In practice, risk often takes precedence over capacity [23,36]. For Llewelyn et al. mental health legislation creates tension for clinical decision makers between prevention of harm and the autonomy of the person experiencing mental health symptoms [23]. Paramedics often make decisions regarding safety and risk with little contextual information regarding the person and their prior contact with the mental health system leading to early decisions that involuntary care is required [21,37]. There is also potential for reattendance by paramedics when the person is not admitted or placed under community mental health team review. In most states and territories, paramedics have access to mental health triage services that are state based, co-response teams (although access may be limited to traditional office hours) and in some circumstances mental health liaisons that are based in the operations centres of the ambulance services are available which can provide further contextual information regarding the patient to assist in clinical decision making. Limited alternate care pathways may lead paramedics to view transport to emergency departments as their only option [38].

### Impact of other legislation

Paramedic practice in relation to mental health may also be complicated by the requirements of other legislation. As an example, Llewelyn et al. [23] note that in Queensland paramedics and police can take persons displaying disturbed behaviour for assessment using Emergency Examination Authority under the *Public Health Act 2005 (Qld).* The criteria for involuntary assessment under the *Public Health Act 2005 (Qld)* are:

(1) The person’s behaviour indicates they are at immediate risk of serious harm;
(2) The risk appears to be the result of a major disturbance in the person’s mental capacity; and
(3) The person appears to require urgent examination or treatment and care [23].

This is less than is required under the *Mental Health Act 2016 (Qld)* and has potential to create a conflict of interest in responding to legislation.

Similarly, section 243 of the Western Australian Criminal Code (7.4.2) states it is lawful for any person to use such force as is necessary in order to prevent a mentally impaired person from doing violence to any person or property [39]. This legislation enables paramedics to use restraint which is not a power granted under the *Mental Health Act 2014 (WA)*. This is problematic as it provides a legal means to extend paramedic powers when responding to people who may be experiencing mental illness.

### Study limitations

The study records how mental health legislation defines the role of paramedics in responding to patients who present with mental health symptoms. Mental health legislation was examined at a specific point in time and as such, the paper provides a snapshot of legislative requirements at the time. An overview of historical changes in state and territory Acts was outside the scope of the study. One Mental Health Act, the *Mental Health Act 2014 (Vic)*, was repealed and replaced by the *Mental Health and Wellbeing Act 2022*. while changes to a second Act (Tas) changed the terminology and exclusion criteria and limited the period that paramedics can detain a patient with mental health symptoms. These changes had limited effect on paramedic role but highlight emerging issues for consideration in management of mental illness.

## Conclusion

This paper has explored the effect of mental health legislation upon expanded paramedic scope of practice in Australia in light of legislative changes associated with the ratification of the CRPD. As with other jurisdictions, Australian mental health legislation does not enact all the legal rights associated with the CRDP. People with mental illness are subject to involuntary detention but the continued use of involuntary detention is tempered by tighter guidelines under which detention can occur. As the states and territories have constitutional responsibility for mental health legislation, each service operates under a different legislative framework. Despite this, there are commonalities in paramedic role with regards to the mentally ill across jurisdictions. In all states and territories except Western Australia and Victoria paramedics can take a person into temporary custody for transport for further assessment. The legislative definition of mental illness; how risk and capacity for informed decision-making is framed, the nature of the powers provided to paramedics all have a part to play in how paramedics conceptualise, interpret and potentially enact their powers under mental health legislation.
